# Photo-EMF Sensitivity of Porous Silicon Thin Layer–Crystalline Silicon Heterojunction to Ammonia Adsorption

**DOI:** 10.3390/s110201321

**Published:** 2011-01-25

**Authors:** Yuriy Vashpanov, Jae Il Jung, Kae Dal Kwack

**Affiliations:** Electrical and Computer Engineering Division, Hanyang Institute of Technology, Hanyang University, 17 Haengdang-dong, Sung-dong-gu, 133-791 Seoul, Korea; E-Mail: jijung@hanyang.ac.kr (J.I.J.)

**Keywords:** gas sensors, porous silicon, heterojunction, photo-EMF

## Abstract

A new method of using photo-electromotive force in detecting gas and controlling sensitivity is proposed. Photo-electromotive force on the heterojunction between porous silicon thin layer and crystalline silicon wafer depends on the concentration of ammonia in the measurement chamber. A porous silicon thin layer was formed by electrochemical etching on p-type silicon wafer. A gas and light transparent electrical contact was manufactured to this porous layer. Photo-EMF sensitivity corresponding to ammonia concentration in the range from 10 ppm to 1,000 ppm can be maximized by controlling the intensity of illumination light.

## 1. Introduction

Porous silicon technologies have many applications in semiconductor technology, optoelectronics, chemical, biological sensors and other fields of science [1–5]. Electrochemical etching of a silicon wafer surface, under different conditions and additional processing, changes the optical and electrical properties of porous silicon layers widely [6]. Changes in electrical and luminescence properties of the porous silicon under gas adsorptions are well-known [7–12]. However, the influence of the gas adsorptions on photovoltaic junction parameters is not known, yet because of the difficulties of obtaining a sensor with a contact which is transparent to both gases and light. Development of a new method in gas analysis on the base of photovoltaic technology by using the contact can be useful for building new type gas sensors.

Photoluminescence of different visible light spectrum in the porous silicon under UV illumination signalize the appearance of quantum nanowires with band gaps larger than crystalline silicon. This means that a heterojunction is formed between the porous silicon and crystal silicon wafer [13]. Photo electromotive force (Photo-EMF) can be measured directly by forming a special light transparent contact on the porous surface. This contact is gas transparent too. With use of this gas and light transparent contact, we can study the properties of heterojunction under gas adsorptions by illuminating visible light. In this paper, we report Photo-EMF gas sensitivity of heterojunction between porous silicon and silicon wafer by using the transparent contact.

## 2. Experimental Section

**Figure 1.**
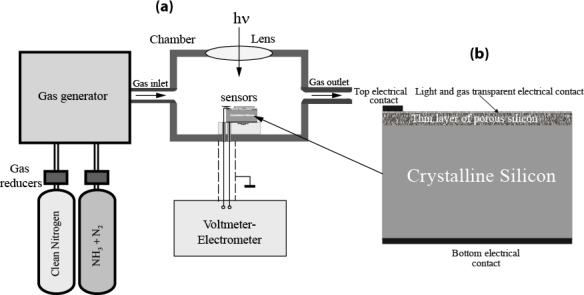
**(a)** Experimental setup and **(b)** cross-section of Photo-EMF sensors structure.

Porous silicon for this study was formed by anode electrochemical etching of (111) oriented p-type silicon wafer with a resistivity of 10 Ωcm at the current density of 10 mA/cm^2^, in a HF-based solution [14]. During the etching process an additional illumination and an ultrasonic processing was applied to the silicon surface. The thickness of porous silicon layers is found from cross-section scanning electron microscopy (approximately 12 μm at 5 min etching time). Thin porous film from aluminum was manufactured on a surface of porous layer [gas and light transparent contact on Figure 1(b)]. This film was transparent to illumination and gas molecules and created electrical contact with porous silicon [top electrical contact on Figure 1(b)]. Top to gas and light transparent contact and bottom electrical contact from aluminum to silicon wafer is manufactured by standard technology [top and bottom electrical contacts on Figure 1(b)].

The photo electromotive forces between top and bottom contacts of samples were studied in a special measuring chamber at room temperature [Figure 1(a)]. We used voltmeter-electrometer of B7-30 type for Photo-EMF registration with double screening. Composition of gas atmosphere was changed by gas generator of GR-645 type by dynamical mixing clean nitrogen and ammonia gases [Figure 1 (a)]. The surface of samples was illuminated at different intensity and wavelengths of light. Illumination level was measured by IL Luminance meter T-10 (Konica Minolta). For spectral measurements a spectrophotometer SF26 was used.

## 3. Results and Discussion

**Figure 2.**
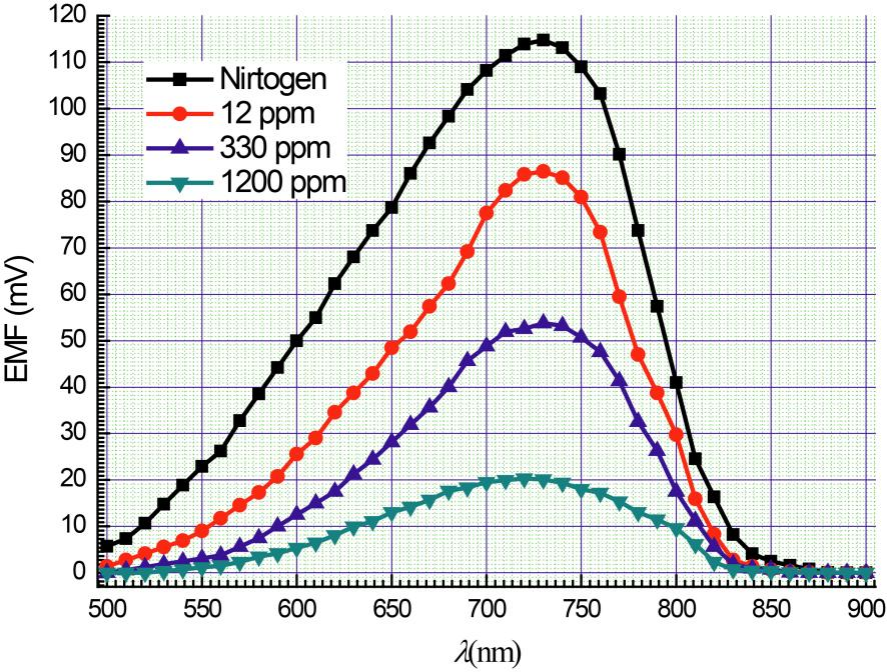
Spectral dependences of Photo-EMF on nitrogen and on different ammonia concentrations.

Electrical voltages (Photo-EMF) between top and bottom contacts [Figure 1 (a)] were registered under illumination of samples. Their magnitudes depended on concentration of ammonia in the measurement chamber. Figure 2 shows spectral dependence of Photo-EMF at an illumination level 200 l× at different concentrations of ammonia in the chamber. A maximal magnitude of Photo-EMF was observed near the wavelength of light of approximately 730 nm (Figure 2). An increase of ammonia concentration in the measurement camber leads to a decrease of the Photo-EMF magnitude. Usually photo-detectors have a maximum photo-response at a wavelength of light corresponding to band gap energy *E*_g_ [15]. In our case the band gap energy of a porous silicon layer equals approximately 1.7 eV (energy of light quantum at wavelength 730 nm [16]).

Manufactured porous silicon layer had red (*λ*_max_ ≈ 780 nm) luminescence under laser illuminations at a wavelength of 441.2 nm. This red luminescence had a half-width at half maximum of approximately 0.2 eV. The luminescence in visible range of spectrum is related to quantum wires [1]. Broad band of luminescence peak demonstrates a different thickness of quantum wires. It means that porous silicon have quantum wires with greater band gap than crystalline silicon. Band gaps of our nanowires system are in the ranges of 1.7 ± 0.2 eV. In our case the heterojunction should be formed between porous silicon and silicon wafer. The electrical contacts had ohmic properties. The light induced heterojunction can be the single reason of occurrence of the photo electromotive force on contacts.

**Figure 3.**
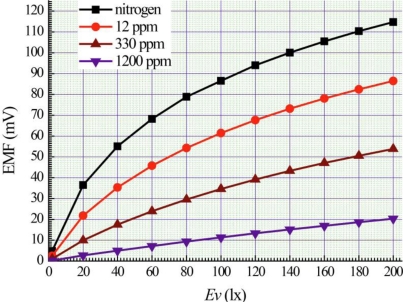
Dependences of maximal magnitudes of Photo-EMF on illumination level under nitrogen and different concentrations of ammonia.

Figure 3 shows the experimental dependences on illumination level of the maximal magnitudes of Photo-EMF under different concentrations of ammonia. The magnitude of Photo-EMF increases with growing illumination level (Figure 3). Adsorption of ammonia in porous silicon layer affected the photo-EMF magnitudes appreciably.

**Figure 4.**
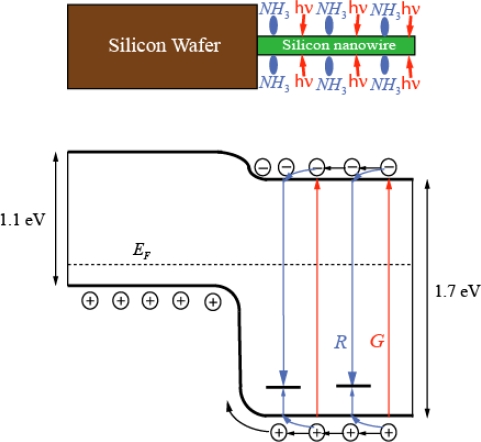
Simplified model of heterojunction energy diagram between silicon nanowire and crystalline silicon wafer under light illumination at wavelength 730 nm and ammonia molecule adsorption.

Measurements between top and bottom contacts of current-voltage characteristics showed typical rectifying behavior. The rectification ratio reached a value of 10^5^. Under small bias the ideality factor was approximately 2. From the data of current-voltage and spectral dependence of Photo-EMF energy band diagram of heterojunction can be found between crystalline silicon wafer and quantum wire (Figure 4).

Illumination of porous silicon layer generate electron-hole pairs *G* in nanowire (generation process is marked in red in Figure 4). These electron-hole pairs recombined through recombination centers (recombination process is marked in blue in Figure 4). A part of holes reach to *p*-type region of crystalline silicon. Then photo electromotive force should appear on heterojunction.

The intensity is inhomogeneous along nanowires. The generation of electron-hole pairs is maximal on top part of the porous layer. However, a physical mechanism of Photo-EMF should be identical with well-known photo electromotive force of light induced heterojunction [17].

Ammonia molecules are adsorbed mainly on surface of wires (Figure 4). Adsorption of ammonia molecules creates new surface levels. A re-charging of levels in quantum wires and electrical micro fields close to polar ammonia molecules can affect on recombination rates of electron-hole pairs. In our case, the ammonia adsorption substantially influences on magnitude of photo-EMF (Figures 2 and 3).

Photo electromotive force can be defined as voltage at the heterojunction under illumination in the absence of a current in a circuit. This voltage can be described by the following formula [17]:

(1) $$U=\frac{\mathrm{AkT}}{e}\;\text{ln}\left(\frac{J_{\mathrm{sc}}}{J_{0}}+1\right)$$

where *J*_sc_ = *ηJ*_ph_, *J*_ph_ is the photocurrent, *η* is the collection efficiency, *J*_0_ is saturation current in darkness, *A* is quality factor of heterojunction [18], *e* is charge of electron, *k* is Boltzmann constant, *T* is temperature. In our case quality factor of heterojunction equals approximately 2.

Increase of light intensity leads to increasing voltage *U* (Photo-EMF) because the photocurrent increases. Decrease of voltage *U* under adsorption of ammonia may be is related to recombination of electron-hole pairs through recombination levels (collection efficiency *η* under adsorption decreases).

**Figure 5.**
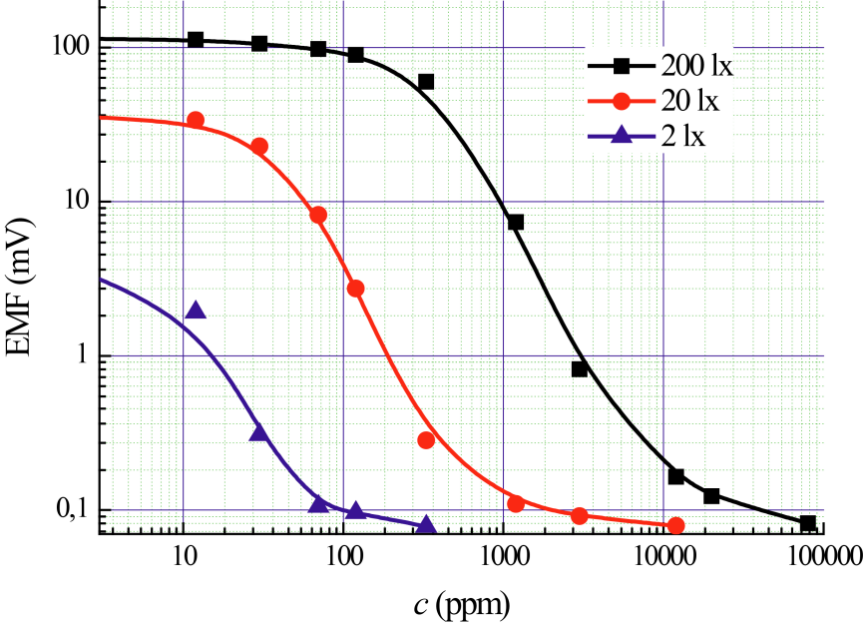
Dependences of Photo-EMF on ammonia concentration under different illumination levels.

Figure 5 shows the concentration dependences of Photo-EMF at different level of illumination. Different levels of Photo-EMF are detected for different ranges of concentration: from 100 ppm to 10,000 ppm at 200 lx, from 10 ppm to 1,000 ppm at 20 lx and from 1 ppm to 100 ppm at 2 lx. In the literature on gas sensor technologies (for example, [19]), no information about controlling sensor’s sensitivity based on the illumination level control is found.

Selectivity properties of gas sensors are very important in practice. Porous silicon has an electrical sensitivity to numerous gases. Probably, the photo-EMF induced by ammonia can be observed for other gases too. There are many publications about humidity sensors on the base of porous silicon (see, for example, [20]). Perhaps, sensitivity of porous silicon to humidity could be reduced as compared with ammonia under the operating temperatures more than 100 °C. Illuminating hetero-junction between porous silicon thin layer and crystalline silicon wafer, with a more powerful and higher irradiance laser beam will be an interesting way of studying photo-adsorption-desorption properties of porous silicon surface to different gases. The photo-EMF method of detecting gases could be used in an automatic gas measuring system in chemical industries related with ammonia and nitrogen molecules.

## 4. Conclusions

Based on our results, we conclude that photo-electromotive force on the heterojunction between a porous silicon thin layer and a crystalline silicon wafer depends on concentration of ammonia in the measurement chamber. Photo-EMF sensitivity corresponding to ammonia concentration in the range from 10 ppm to 1,000 ppm can be maximized by controlling the intensity of illumination light. The measuring photo-EMF in porous silicon based gas sensor will open a new way of building gas sensors.
